# Supplementary material for: The interactome of intact mitochondria by cross-linking mass spectrometry provides evidence for coexisting respiratory supercomplexes
Source: Mol Cell Proteomics. 2017 Dec 8;17(2):216–32. doi: 10.1074/mcp.RA117.000470 (PMC5795388; doi:10.1074/mcp.RA117.000470)
Supplement: Supplemental Data [file supp_RA117.000470_133922_0_supp_23978_fzffwf.zip › spectra_annotation/mito_DR_spectra_annotation/209-1-21-1-15-1.pdf]

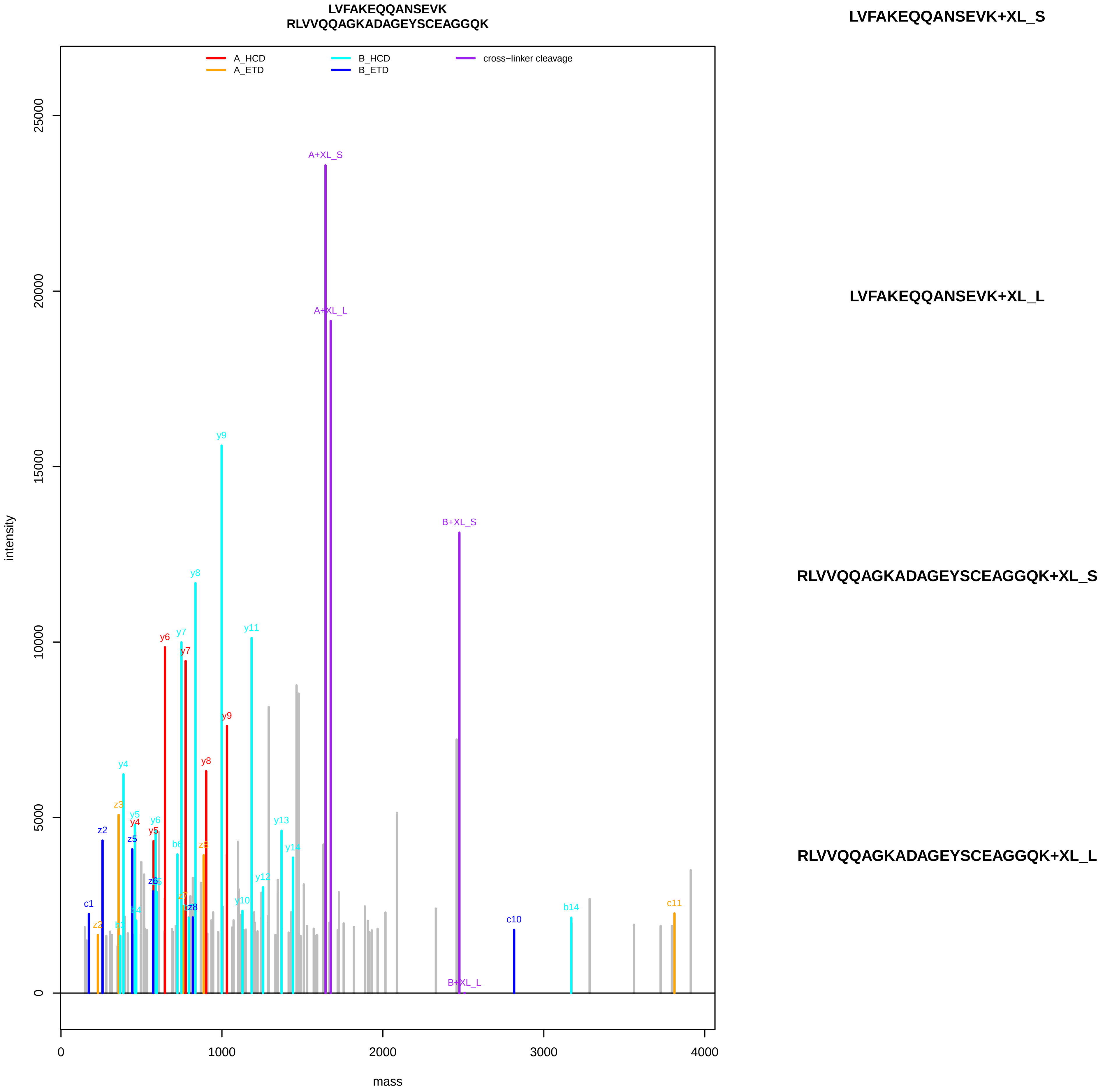
LVFAKEQQANSEVK
RLVVQQAGKADAGEYSCEAGGQK
A_HCD
A_ETD
B_HCD
B_ETD
cross−linker cleavage
A+XL_S
A+XL_L
B+XL_S
B+XL_L
y9
y8
y11
y7
y6
y7
y9
y8
y4
z3
y5
y4
y5
y6
z2
z5
y13
y14
y12
c1
z6
y10
z8
c10
b14
c11
intensity
mass
0
5000
10000
15000
20000
25000
0
1000
2000
3000
4000
LVFAKEQQANSEVK+XL_S
LVFAKEQQANSEVK+XL_L
RLVVQQAGKADAGEYSCEAGGQK+XL_S
RLVVQQAGKADAGEYSCEAGGQK+XL_L
